# The Role of* Anethum graveolens* L. (Dill) in the Management of Diabetes

**DOI:** 10.1155/2016/1098916

**Published:** 2016-10-18

**Authors:** Mohammad Taghi Goodarzi, Iraj Khodadadi, Heidar Tavilani, Ebrahim Abbasi Oshaghi

**Affiliations:** ^1^Department of Clinical Biochemistry, Medical School, Hamadan University of Medical Sciences, Hamadan, Iran; ^2^Research Center for Molecular Medicine, Hamadan University of Medical Sciences, Hamadan, Iran

## Abstract

*Aim*. There is evidence that* Anethum graveolens* (AG) has been used for centuries in Asian traditional medicine, and its constituents have useful effects on the control and management of diabetes and cardiovascular disorders. AG has many useful effects, including hypolipidemic and hypoglycemic effects, and it has been reported to reduce the incidence of diabetic complications. It acts mainly by affecting antioxidant capacity and change in some genes in glucose and lipid pathways. The aim of the present paper was to summarize pharmacological effects of AG in the management of diabetes.* Methods*. To prepare this review, a pharmacological and phytochemical literature survey was performed using Scopus, PubMed, and Web of Science. Also, some historical and ethnopharmacological literature sources were used.* Results*. This review plans to provide readers with an assessment of the pharmacological effects of AG, especially in diabetes.* Conclusion*. The paper highlights the therapeutic effects of AG which would aid in supporting their safe use in the management of diabetes and cardiovascular diseases.

## 1. Introduction

Diabetes mellitus is a chronic metabolic disorder that is distinguished by hyperglycemia, underutilization of blood glucose, and defects in the metabolism of macronutrients such as carbohydrates, fat, and protein, secondary to the disturbance in insulin action [1, 2]. Type 1 diabetes is caused as a result of insulin deficiency due to failure of pancreatic beta-cells. Therefore, diabetic patients need exogenous insulin injection, whereas patients with type 2, known as insulin independent, do not respond to insulin and consequently can be managed by lifestyle changes. Type 2 diabetes constitutes 90% of the diabetic cases in the world [3]. Next to cancer and heart disease, diabetes mellitus is the third most life-threatening disorder which has a high rate of morbidity and mortality worldwide. Prevalence of this disorder is quickly rising in every part of the world [4]. As a result, it is projected to affect 552 million people in 2030, rising sharply from 336 million in 2011 [3]. Signs of type 1 and 2 diabetes may include hyperglycemia, abnormal thirst, repeated urination, weight loss and severe hunger, nausea and vomiting, mood changes, and severe weakness and tiredness [1]. The purpose of this review was to identify* Anethum graveolens* L. (AG) as a valuable herbal plant, originating from Asian traditional medicine, in the management of diabetes. Conducted studies could allow representing the potential applications for future research. To complete the assumed task, traditional sources regarding the usage and the carried out investigation on* Anethum graveolens* L. are collected and explained. Essential assessments of the documented bioactive components particularly in terms of their correlation with recognized traditional use and current pharmacological knowledge are discussed. Phytochemical studies, antidiabetic properties, suggested mechanism, and the main compounds of AG that may be responsible for useful effects of AG are described too.

## 2. Materials and Methods

To prepare this review a pharmacological and phytochemical literature survey was performed using Scopus, PubMed, and Web of Science.

## 3. Results and Discussion

### 3.1. Blood Glucose and Lipid Profile in Diabetic Patients

Studies have shown that blood lipid peroxidation markedly rises in diabetes due to vascular injury, which is provoked by hyperglycemia and free radicals. This peroxidation can further aggravate the progress of diabetic complications and is thought to happen as a result of defects in insulin secretion by pancreatic *β*-cells. Diabetes also leads to an impaired lipid profile, including low levels of high-density lipoprotein (HDL) cholesterol and hypertriglyceridemia and raised oxidative stress [30, 31]. Diabetes also raises the risk of heart disease two- to fourfold. Therefore, uncontrolled hyperlipidemia leads to premature cardiovascular diseases (CVDs). CVD is the most common complication in diabetes, which is a common reason for premature mortality in diabetic patients. As a result, to reduce the risk of CVD, many studies potentially recommended control of dyslipidemia and hyperglycemia [1, 32–34].

In addition to dyslipidemia, hyperglycemia is known as a major risk factor for diabetic complications. Protein glycation is involved in the progress of complications such as retinopathy, neuropathy, nephropathy, dermopathy, and atherosclerotic lesions [1, 33, 35–37]. In the chronic state, excess glucose binds to free amino groups in tissue or blood proteins and body fluid and disturbs their functions [38–40]. This nonenzymatic reaction leads to formation of advanced glycosylation end products (AGEs). Glycation reaction leads to alteration in the structure and function of biomolecules particularly proteins. AGE formation is known as a key element in diabetes complications [41–46].

### 3.2. Free Radicals in Diabetes

In diabetic patients, reactive substances such as hydroxyl radicals (^•^OH), hydrogen peroxide (H_2_O_2_), lipid peroxyl radicals (LOO^•^), superoxide anion (O_2_^•−^), and peroxyl radicals (ROO^•^) are mainly generated during the developing stage of diabetes. The reaction of these reactive species with polyunsaturated fatty acids (PUFAs) causes lipid peroxidation which is consequently implicated in early diabetes complications, especially premature cardiovascular disease [47, 48].

Enzymatic antioxidant mechanisms play an important role in the elimination of free radicals. For instance, superoxide dismutase (SOD) is known as one of the main endogenous enzymes that removes O_2_^−^ and produces H_2_O_2_, which is then processed to water by the activity of catalase (CAT) and glutathione peroxidase (GPx). Hence, SOD can work as the chief protector against O_2_^•−^ and it inhibits production of free radicals. CAT is an important enzyme involved in detoxification of hydrogen peroxide to water and oxygen. On the other hand, glutathione S-transferase (GST) and glutathione peroxidase (GPx) are other chief antioxidant enzymes [47, 49, 50]. GPx decreases free radical damage by preventing the production of extremely cytotoxic species, for instance, lipid peroxides and other organic hydroperoxides. GST is therefore believed to preserve the balance of cellular redox conditions [47, 49]. Lipid peroxidation naturally occurs on a small level in the body and plays a key role in diabetes and cardiovascular diseases pathogenesis. The reactive oxygen species such as hydroxyl radical and hydrogen peroxide attacks polyunsaturated fatty acids and causes lipid peroxidation. Iron ion and hydrogen peroxide in a known process, called Fenton reaction, lead to formation of hydroxyl radical which is a potent oxidative agent [15]. Oral administration of many herbal plants leads to a marked rise in the activities of SOD, CAT, GSH, GST, and GPx in diabetes [51, 52].

### 3.3. Diabetes Treatment

There are different ways for treatment and control of diabetes, including administration of insulin or hypoglycemic drugs, dietary control, and exercise [53, 54]. Furthermore, chemical drugs generally have low efficacy over time, are too expensive, and show severe side effects such that some patients cannot tolerate prolonged treatment or taking the high dosages of these drugs. In contrast, herbal medicines are regarded as potential sources for medication due to their useful effects, negligible side effects in clinical trials, and low cost [53–59].

### 3.4. Safety and Side Effects of Chemical Diabetic Medicines

People use different medicines such as sulfonylureas (glimepiride, glipizide, and glyburide), biguanides (metformin), thiazolidinediones (pioglitazone), alpha-glucosidase inhibitors (acarbose), meglitinides (nateglinide), and dipeptidyl peptidase 4 inhibitors (Januvia, Onglyza) for control and treatment of hyperglycemia. However, side effects can be seen in all diabetes drugs' usage. More than 10 to 20 percent of diabetic patients are known to eventually stop taking their medications because of the side effects. Some of the diabetes medicines can also cause hypoglycemia which is a harmful and potentially lethal side effect. Additional side effects of several antidiabetic drugs are weight gain, increased risk for fractures especially in women, and gastrointestinal disorders such as abdominal pain, gassiness, vomiting, nausea, diarrhea, and bloating. Edema and increased level of low-density lipoprotein cholesterol (LDL-C) were also reported by many patients. Uncommon side effects include congestive heart failure, allergic reactions, and anemia, which can be induced by chemical diabetic medicines [30, 31].

### 3.5. Role of Herbal Medicine in the Treatment of Diabetes Mellitus

Nowadays, many physicians prescribe insulin, thiazolidinediones, sulphonylureas, *α*-glucosidase inhibitors, and so forth, for treatment and control of diabetic complications [75]. Nevertheless, since adverse effects of these medicines are arguable, there is a need for novel agents in diabetes treatment. In this respect, some herbal medicines due to their lower side effects, low costs, useful properties such as antihyperglycemic, antihyperlipidemic, and antioxidant effects, and especially their natural origin have been proposed for potential management of diabetes [76, 77]. However, because of unknown mechanisms, only a small number of plants have been used for the management of diabetic complications [78]. Herbal plants contain many chemical components such as flavonoids (30%), terpenoids (17%), saponins (9%), polyphenols (6%), alkaloids (5%), tannins (4%), polysaccharides (3%), and miscellaneous compounds (17%), which are known to have remedial properties [78]. Interestingly, the World Health Organization (WHO) reported that about 80% of the world's people presently use herbal plants for treatment of different diseases [79]. Although many plants have been suggested for treatment of diabetes due to their antidiabetic properties, in this review, we will attempt to open a new window toward application of AG and its constituents in the management of type 2 diabetes.

### 3.6. Botanical Profile and Taxonomy of AG

**Figure 1:**
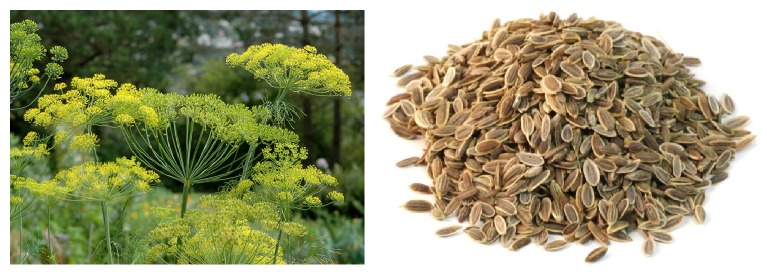
Plant and seed of AG.

The generic name of “*Anethum*” comes from the Greek word “anethon” and the colloquial name of dill is derived from the Old Norse word “dilla” that possibly means “to soothe.” AG is the only species of the genus* Anethum*, still classified by several botanists related to* Peucedanum* genus as* Peucedanum graveolens* (L.) [80]. AG is an annual vertical plant with 50–150 cm stem belonging to the Apiaceae family. The fruits of AG are brown, flat, tiny, and oval in shape (Figure 1). This plant is cultivated in Mediterranean countries, in Asia, and in Europe. AG contains 36% carbohydrates, 15.68% proteins, 14.80% fiber, 9.8% ash, and 8.39% moisture as well as essential oils, fatty oil, minerals, and vitamins [80].

### 3.7. Traditional Uses of AG

AG is used in the traditional herbal medicine for the management and prevention of digestive disease, breath problem, motivation of lactation, and also reduction of cholesterol and glucose. Currently, many studies have established these properties; also, AG is recently known as anticancer, antimicrobial, antigastric irritation, anti-inflammatory, and antioxidant agent. In this respect, dill is produced as a hypolipidemic drug (*Anethum* tablet) in Iran which consists of* Anethum graveolens* (68%),* Cichorium intybus* (5%),* Fumaria parviflora* (5%), and lime (Citrus aurantifolia) (4%) [81].

### 3.8. Hypolipidemic and Hypoglycemic Properties of AG

Administration of different extractions of AG seed and leaf, as well as its essential oil, in diabetic models significantly reduced triglycerides, total cholesterol, low-density lipoprotein cholesterol (LDL-C), very-low-density lipoprotein cholesterol (VLDL-C), and glucose levels, whereas it increased HDL-C level [80]. On the other hand, many studies reported that AG has hypoglycemic and antioxidant activity [80]. The antioxidant activity of AG is due to its phenolic proanthocyanidins and flavonoids constituents [24]. It has also been shown that the extract from AG flowers had more antioxidant activity than either its seed or leaf extract [80]. Duration of treatment in different experiments was variable; for example, Koppula and Choi [17] used only single dose for one hour, while the treatment period in Mansouri et al.'s [11] study was 12 weeks. However, almost all of the experiments show that one month is an appropriate time for survey of antidiabetic properties of dill.

To survey the effects of AG on dyslipidemia, different animals were used. The rabbit is known as an animal model that has been commonly used for the study of hypercholesterolemia. Only few studies have been done on rabbits, whereas rats are an optional model for diabetes studies [7]. Therefore, as mentioned in Table 1, most of the studies used diabetic rats.

Some primary mechanisms have been proposed for hypolipidemic effects of AG. Inhibition of intestinal cholesterol absorption, binding to bile acids in the intestine, an increase in fecal excretion, and increasing production of bile acids might be the main mechanisms by which AG exerts its antidiabetic functions [80]. Yazdanparast et al. [24, 82] proposed that some components of AG such as carvone, limonene, or *α*-phellandrene are responsible for the hypolipidemic properties of AG, likely through reducing acyl CoA carboxylase or 3-hydroxy-3-methylglutaryl-CoA (HMG-CoA) reductase, the key enzymes in fatty acid and cholesterol metabolism. Other studies have suggested that AG components might rise liver LDL receptors, decrease fatty acid synthesis, and impact lipoprotein homeostasis mainly through improvement in lipoprotein metabolism [24]. Furthermore, some studies suggest that AG may improve the lipid profile by its antioxidant properties [10]. We have also observed that administration of 100 and 200 mg/kg of AG tablet and hydroalcoholic extract of AG in hypercholesterolemic hamsters resulted in significantly reduced HMG-CoA reductase activity and mRNA levels [83]. For the first time, we also showed that AG normalized the lipid profile by decreasing HMG-CoA reductase in the hypercholesterolemic hamsters and type 2 diabetic rats [83]. We have also shown that AG increased LDL receptor level in the liver resulting in stimulation of cholesterol clearance from the bloodstream (unpublished data). The results of Abbasi Oshaghi et al.'s [84] study showed that AG significantly increased cholesterol-7-alpha hydroxylase activity, the rate-limiting enzyme in bile acids biosynthesis [10]. Some studies have also reported that rutin and quercetin (components of dill) may decrease serum cholesterol and LDL-C levels and decrease liver cholesterol level. Other studies have also reported that quercetin reduces HMG-CoA reductase activity [24]. Histopathological experiments of Abbasi Oshaghi et al. [84] showed that treatment of diabetic rats with different extracts of AG normalized lipid deposits in the liver, pancreas, and heart. HPLC analysis showed that AG has high amounts of quercetin which may be responsible for the suppression of HMG-CoA reductase activity [10]. Numerous studies that have previously shown the antidiabetic effects of AG are listed in Table 1. Chemical compounds of AG extracts especially flavonoids, terpenoids, alkaloids, tannins, and phytosterols are probably responsible for its hypoglycemic effects [61]. We found that different extracts of AG reduce AGEs formation [85, 86]. Our study also showed that AG at the dose of 300 mg/kg had potential antioxidant and hypolipidemic effects [86]. Madani et al. [28] reported significant decline in fasting blood glucose level with 300 mg/kg hydroalcoholic extract of* Anethum graveolens*. Doses of AG up to 2 g/kg in a short period of time did not cause any death in animal models. Also, the AG doses up to 1000 mg/kg did not cause any toxic effect in short time experiments [87]. However, 50 mg/kg/day is the minimum and 1 g/kg/day is the maximum tolerated dose in a 45-day period [87]. Although different doses of AG were used in animal models, Ahmadi Mahmoudabadi [22] reported that the effective dose for metabolic properties is 300 mg/kg in animal models. Different studies showed that both high and low doses of AG have hypoglycemic and hypolipidemic effects. The doses less than 50 mg/kg could be known without adverse effect in rats and mice [87]. However, Piri et al. [18] demonstrated that 3-week administration of* Anethum* extract at the doses of 50, 100, and 200 mg/kg normalized blood lipid, and the change in blood glucose was not significant. The safe and effective dose in human studies is reported as 650 mg/kg [26]. In an* in vitro* study, Abbasi Oshaghi et al. [86] found that different extracts of AG at the concentrations of 0.032, 0.065, 0.125, 0.25, 0.5, and 1 mg/ml reduced AGEs formation; fructosamine level and carbonyl groups, also, raised thiol groups in type 2 diabetic animals. The components of AG are responsible for its antiglycation properties. Oxidation of carbonyl and thiol groups on blood proteins significantly reduces by* Anethum* in a dose dependent manner [28].

According to the study conducted by Adisakwattana et al. [88], polyphenolic compounds in plant extract may play a major role in the inhibition of AGEs formation. AGEs are recognized to contribute to the production of oxidative stress and inflammation, which are related to the diabetes complications. It has been reported that administration of AGE-rich diets in an animal model is associated with rising of blood and tissue AGEs and the conditions like atherosclerosis [89]. Mobasseri et al. [90] showed that administration of AG powder in type 2 diabetic patients reduced fasting blood glucose and normalized insulin resistance and lipid profiles. As mentioned above, AG is rich in antioxidant compounds; therefore, antioxidant and flavonoid components of AG probably are able to repair damaged *β*-cells and insulin secretion. It has been shown that AG significantly increases total antioxidants in the pancreas and also regenerates histopathological changes [84]. Therefore, AG with antioxidant activity likely increases insulin synthesis. Some researchers such as Madani et al. [28] and Mishra [6] reported that AG reduced blood glucose via the increase of insulin secretion. Takahashi et al. [91] have shown that AG extract normalized lipid profile in diabetic obese mice by activating peroxisome proliferator-activated receptor-*α* (PPAR-*α*) and increasing fatty acid oxidation-related genes expression.

Our colorimetric and HPLC analysis also showed that dill had a high amount of quercetin, phenol, flavonoid, tannin, saponin, and alkaloid [84]. All of these agents have hypoglycemic and hypolipidemic properties. Therefore, we can conclude that components of dill are responsible for its metabolic effects. Mishra [6] reported that aqueous extract of AG has a high amount of carvone, which is accountable for its antidiabetic activity. Carvone is able to cause decline in plasma glycoprotein components and motivate insulin secretion. The ethanolic extract of AG which is prepared in India showed very low antidiabetic properties [6]. On the other hand, Mudassir et al. [92] reported that the prepared ethanolic extract of AG in Pakistan significantly reduced blood glucose (34% reduction). They declared that hypoglycemic effects of AG might be due to the inhibition of intestinal glucose absorption [92].

In our laboratory, we have previously shown that AG has potential antioxidant activity [85]. Our results are in agreement with the results of a previous study by Bahramikia and Yazdanparast [20], indicating DPPH radical scavenging activity of AG. In our study, 1 mg/ml of AG water extract showed 96.04% scavenging of H_2_O_2_, whereas only 27.26% radical scavenging activity was reported for AG ethanolic extract [86]. AG also showed Fe^2+^ ion chelation which is known as a defense mechanism against oxidative damage by declining free radical production via Fenton reaction. In this reaction, iron(II) is oxidized by H_2_O_2_ to create potential free radicals such as hydroxyl radical. Nitric oxide scavenging activity has also been proposed for AG [20]. The results of a study by Bahramikia and Yazdanparast [20] showed that crude extract of AG exhibited a moderate NO scavenging activity. In line with this observation, we showed a nitric oxide scavenging activity of AG similar to the known radical scavengers, ascorbic acid and butylated hydroxytoluene (BHT) [86]. Interestingly, we showed that NO scavenging activity of AG is comparable with the activity of ascorbic acid and/or BHT. We showed that a 1 mg/ml solution of dill water extract has 90.41% NO scavenging activity which was much greater than that observed (78.49%) for ethanolic extract of AG by Orhan et al. [93]. We have also detected a greater antioxidant activity for AG water extract compared with methanolic extracts [85]. Interestingly, the antiradical and antioxidant properties of AG in all tests were similar to ascorbic acid and BHT [80]. The results of many studies established the notion that the particular parts of dill are often discriminated by significant diversity in their constituents' chemical compounds. It has been reported that ethanolic extract of AG has very low antidiabetic properties when compared with the aqueous extract [6]. However, Mishra reported that ethanolic extract of AG has hypoglycemic effects [6]. Concordant with the hypoglycemic function of AG ethanolic extract, other studies have reported that this extract significantly reduces blood lipids [24, 82]. Interestingly, we showed that different extracts of AG significantly reduced glucose level, but the reduction by water or hydroalcoholic extract was more as compared with the methanolic extract [86]. Furthermore, with review of the literature, we can find that different preparations such as hexane, ethanolic, water, and hydroalcoholic extracts of seed and leaf have been used in different studies (Table 1). However, the hydroalcoholic extract showed stronger antidiabetic effects compared with other extracts.

### 3.9. Chemical Composition

There is some diversity in the dill composition in different countries; for example, American dill oil has a high amount of alpha-phellandrene, while carvone and limonene are the main constituents of Asian and European dill [94]. In addition to essential oils especially carvone, limonene, and alpha-phellandrene, dill also contains fatty oil, proteins, carbohydrates, moisture, fiber, ash, vitamins (A and niacin), and mineral elements (calcium, potassium, magnesium, phosphorus, and sodium). This plant is known as a rich source of flavonoids, phenolic compounds, saponins, cardiac glycosides, and terpenes [80].

Jana and Shekhawat [80] reported that there are several volatile components of AG seeds and herb. For instance, carvone is the main odorant of AG seed and limonene, *α*-phellandrene, myristicin, and dill ether are the most vital odorants of AG herb. Larijani et al. [63] also reported that seeds and leaves of AG have different components and consequently have different therapeutic effects. Different parameters such as cultivating area, genotype, and environmental factors have huge effects on the composition of AG extracts [80]. Furthermore, Reichert et al. [95] reported that the different parts of AG have different compounds and these compounds may change depending on the stage of plant growth. For example, Sefidkon [96] reported that *α*-phellandrene and limonene are the major constituents of dill oil, while Singh [64] showed that carvone, dill apiole, limonene, trans- and cis-dihydrocarvone, and linalool are the main constituents. Consequently, the different components which are reported by the different studies also depend on the different plant growth stage, cultivating area, and the particular part of the plant that is used. Different compounds of AG have been described in the different published reports which are listed in Table 2.

### 3.10. Adverse Effects

However, it has been shown that AG administration is safe, and it may rarely lead to allergic reactions, vomiting, diarrhea, oral pruritus, urticaria tongue, and throat swelling [97]; furthermore, it is not suggested in pregnancy [97]. Mirhosseini et al. [98] reported that AG did not have any side effects, as compared with gemfibrozil, in treated patients. Other clinical trial studies did not report the side effect of AG [11, 13, 26, 27].

## 4. Conclusion

Herbal medicines have multiple key components such as flavonoids, terpenoids, saponins, polyphenols, tannins, alkaloids, and polysaccharides that have their individual remedial properties. The use of herbal medicines is not a novel matter. Herbal medicines especially AG have a lot of useful properties including antihyperlipidemic, antihypercholesterolemic, antidiabetic, anticancer, antioxidant, antistress, antisecretory, cardioprotective, antispasmodic, and diuretic effects. Moreover, epidemiologic experiments reported a converse relationship between AG consumption and risk of diabetes and CVD progression. Recent literature strongly supports the suggestion that consumption of AG has a significant antidiabetic effect in both humans and animals. According to the reported antidiabetic effects of dill, it can be suggested for the management of diabetic patients. However, the diverse preparations, dose of AG, period of AG consumption, and interaction with other drugs must be normalized. More studies are required to recognize the specific constituents of AG that are accountable for most of its valuable properties. Although all components of AG have antioxidant, hypoglycemic, and hypolipidemic effects, these components are at low levels in dill and possibly have synergic effects. Moreover, the findings suggest that activation of LDL-R, PPAR-alpha, and other FA oxidation-related genes and also inhibition of HMG-CoA reductase contribute to the hypolipidemic effects of AG.

## Figures and Tables

**Table 1:** Antidiabetic effects of AG in different studies.

Reference	Preparation	Study model and duration	Pharmacological properties
[5]	Alkaloid extracted from AG	Hypercholesterolemic rabbits, 4 weeks	(i) Hypolipidemic effect

[6]	Ethanolic extract of AG seed	Alloxan induced diabetic mice, 15 days	(i) Hypoglycemic effects

[7]	Hydroalcoholic extract of AG	Hypercholesterolemic rabbits, 3 days	(i) Significant reduced glucose levels, LDL-C, TC, AST, ALT, and fibrinogen (ii) The change in ApoB, factor VII, nitrite, and nitrate was not significant

[8]	Hydroalcoholic extract of AG	Hypercholesterolemic rabbits, 3 days	(i) Significantly declined TC, LDL-C, AST, ALT, and fibrinogen

[9]	Hexane extract of AG seed	High-fat-diet-induced hyperlipidemia and diabetic rat, 4 weeks	(i) Normalized blood lipid and glucose (ii) Increased blood adiponectin levels (iii) Significantly motivated FA oxidation by induced expression of FA oxidation-related genes (especially with activation of PPAR alpha) and also inhibited TG accumulation

[10]	Methanolic extract of AG	*In vitro *	(i) Antioxidant activity

[11]	Hydroalcoholic extract of AG	Patient with metabolic syndrome, 12 weeks	(i) Significantly reduced TG

[12]	Combined carnitine and hydroalcoholic extract of AG	Rat, 21 days	(i) AG does not improve serum lipids profiles

[13]	AG along with aerobic training	Type 2 diabetic patients, 4 weeks	(i) Hypoglycemic effects (ii) Hypolipidemic effect

[14]	AG leaves powder	Hyperlipidemic patients, 4 weeks	(i) Hypolipidemic effect

[15]	Ethanolic extract of AG	Hepatotoxicity induced by carbon tetrachloride in albino rats, 21 days	(i) Antioxidant and hepatoprotective activity

[16]	Ethanolic extract of AG	*In vitro*	(i) Antioxidant and antiradical and antityrosinase activity (ii) Inhibited lipid peroxidation

[17]	Aqueous extract of AG	Normal and scopolamine-induced amnestic rats, single dose	(i) Significant antistress and antioxidant memory enhancing and activity

[18]	AG tablet	STZ-induced diabetic mice, 21 days	(i) Significantly declined body weight, TG, and LDL-C and increased HDL (ii) The change of blood glucose was not significant

[19]	Ethanolic extract and oil of AG	*In vitro*	(i) Antioxidant activity (ethanolic extract had higher activity than essential oil)

[20]	AG powder	Hyperlipidemic rats, 30 days	(i) Significant antioxidant and radical scavenging activity (ii) Hypolipidemic effect

[21]	AG powder and essential oil	Hypercholesterolemic rat, 2 weeks	(i) Hypolipidemic effect

[22]	Hydroalcoholic extract of AG	Type 1 diabetic rats, 10 days	(i) Normalized blood lipids (ii) Hypoglycemic effects (similar to glybenclamide)

[23]	Hydroalcoholic extract of AG	Corticosteroid induced diabetic rats, 15 days	(i) Significantly declined both plasma glucose and insulin levels (ii) Antioxidant activity

[24]	Ethanolic extract of AG	Hyperlipidemic rats, 30 days	(i) Probably inhibited activity of HMG-CoA reductase (ii) Hypolipidemic effect

[25]	Hydroalcoholic extract of AG seed extract	Rat, 6–168 hrs	(i) Antioxidant and antiradical activity (ii) Significantly reduced liver enzymes

[26]	*Anethum* tablet twice daily (650 mg)	Hyperlipidemic patients, 6 weeks	(i) Normalized blood lipids

[27]	*Anethum* tablet twice daily (650 mg)	Hyperlipidemic patients, one month	(i) Hypolipidemic effect

[28]	Hydroalcoholic extract of AG	Alloxan induced diabetic mice, 48 h	(i) Hypolipidemic effect (ii) Hypoglycemic effects

[29]	AG tablet	Hyperlipidemic patients, 1 month	(i) Hypolipidemic effect

**Table 2:** Components of AG in different studies.

References	Preparation	Components
[60]	Essential oil	Carvone, *α*-pinene, *α*-phellandrene, myrcene, dillapiole, anethole, p-cymene, limonene, dill ether, trans-dihydrocarvone, cis-dihydrocarvone, camphor

[61]	Dill seed oil	Carvone, limonene, gamma-terpinene, m-cymene, apiol, myristicin, trans-dihydrocarvone

[62]	Dill seed oil	Glycosides, reducing sugar, tannins, saponins, flavonoids, steroids, flavonosides, terpenoids

[63]	Dill seed oil	*α*-Phellandrene, sabinene bicycle, trans-caryophyllene, beta-santalene, eugenol, pentadecane, dill apiole, hexadecanol, methyl undecanoate, limonene, eudesmol, n-octane, mefranal, D-carvone, cyclohexasiloxane

[64]	Essential oil	*α*-Thujene, cyclohexane, *β*-myrcene, *β*-phellandrene, grandisol, thujyl alcohol, carvone, bis-1,2-benzenedicarboxylic acid, limonene

[65]	Essential oil	*α*-Pinene, *β*-pinene, *α*-thujene, *β*-phellandrene, *α*-cadinol, sabinene, (Z)-anethole, *α*-phellandrene, limonene, myristicin, *γ*-terpinene, *β*-myrcene, dimethyl phencone, phencone, linalool, dill ether, p-cymene, (Z)-dihydrocarvone, (E)-dihydrocarvone, (E)-carveol, carvone, apiole, phthalide isomer, D-germacrene, epi-bicyclosesquiphellandrene

[66]	Essential oil	*α*-Pinene, *α*-phellandrene, *α*-thujene, *β*-phellandrene, *β*-myrcene, carvone, dill ether, n-eicosane, limonene, n-eicosane, terpinolene, trans-dihydrocarvone, sabinol, apiole

[67]	Essential oil	Limonene, dill ether, *α*-thujene, sabinene, *β*-myrcene, *α*-pinene, p-cymene, *α*-phellandrene, cis-dihydrocarvone, carvone, cadinol, trans-dihydrocarvone, *α*-copaene, *γ*-muurolene, neophytadiene, n-nonadecane, n-docosane, n-heneicosane, n-tricosane, n-tetracosane, n-eicosane, n-pentacosane, n-heptacosane, n-hexacosane

[68]	Essential oil	Limonene, linalool, D-carvone, carvacrol, sabinene, dihydrocarvone, *β*-myrcene, *ρ*-cymene, *γ*-terpinene, E-limonene oxide, estragole, *α*-pinene, terpineol, cumin aldehyde, *β*-caryophyllene, D-germacrene, *α*-phellandrene, thymol, dill apiole, myristicin

[69]	Dill flower extract	Gentisic acid, kaempferol, gallic acid, catechin, chlorogenic acid, luteolin, epicatechin, p-coumaric acid, sinapic acid, benzoic acid, p-anisic acid, myricetin, quercetin, caffeic acid, total phenolic acid, apigenin, proanthocyanidins, total flavonoid

[70]	Dill seed oil	cis-Dihydrocarvone, farnesene, m-carveol, dihydrocarvone, D-carvone, myristicin, cis-dihydrocarveol, 3,5-dimethylcyclohexen-1-one, limonene, diplaniol, 1,2-diethoxyethane, *α*-phellandrene, *β*-myrcene, limonene, ethylacetate, sabinene, *α*-humulene, carvone, cis-carvone

[21]	Dill weed oil	Dill ether, limonene, *α*-phellandrene, *α*-pinene

[71]	Dill oil and acetone extract	*α*-Pinene, *α*-ylangene, *α*-thujene, foeniculin, sabinene, myrcene, p-cymene, sylvestrene, isocaryophyllene, terpinolene, linalool, trans-carveol, camphor, dill ether, cis-dihydrocarvone, trans-dihydrocarvone, undecane, isodihydrocarveol, geranyl acetate, carvone, limonene, *α*-humulene, D-germacrene, *β*-caryophyllene, *β*-selinene, trans-guaiene, *α*-cadinene, *β*-bisabolene, trans-isocroweacin, dill apiole, apiole, elemicin, trans-anethole, eugenol, p-anisaldehyde, lauric aldehyde, trans-bergamotene, nonadecanone, myristicin, trans-muurolol, heptadecane, neophytadiene, palmitic acid, linoleic acid, *γ*-terpinene

[72]	Essential oil	*α*-Thujene, *α*-phellandrene, camphene, sabinene, *α*-pinene, myrcene, p-cymene, limonene, *β*-phellandrene, (Z)-*β*-ocimene, (E)-*β*-ocimene, terpinolene, *α*-p-dimethyl styrene, carvone, bornyl acetate, D-germacrene, *β*-bisabolene, myristicin, T-cadinol, phthalide isomer, farnesol, apiole, n-octadecane, n-heptadecane, n-nonadecane

[73]	Essential oil	Glycosides, saponins, tannins, terpenoids, steroids, flavonoids, phlobatannin, cardiac glycoside, anthraquinone

[74]	Fruit essential oil	Carvacrol, D-carvone, cis-carveol, trans-carveol, cis-dihydrocarvone, trans-dihydrocarvone, limonene, terpinene-4-ol, d-dihydrocarveol, l-dihydrocarveol, a- and g-terpinene, thymol, a-phellandrene, b-terpineol, p-cymene
